# Supplementary material for: Curated and harmonised transcriptomics datasets of interstitial lung diseases
Source: Data Brief. 2025 Oct 14;63:112139. doi: 10.1016/j.dib.2025.112139 (PMC12581653; doi:10.1016/j.dib.2025.112139)
Supplement: Supplementary file 1 [file mmc1.zip › Supplementary_material/RNA-seq/fastQC reports/GSE169500/TRIMMED_SRR14057114_fastqc.html]

TRIMMED\_SRR14057114.fastq.gz FastQC Report 

FastQC Report

Sat 3 Dec 2022  
TRIMMED\_SRR14057114.fastq.gz

## Summary

- Basic Statistics
- Per base sequence quality
- Per sequence quality scores
- Per base sequence content
- Per sequence GC content
- Per base N content
- Sequence Length Distribution
- Sequence Duplication Levels
- Overrepresented sequences
- Adapter Content

## Basic Statistics

| Measure | Value |
| --- | --- |
| Filename | TRIMMED\_SRR14057114.fastq.gz |
| File type | Conventional base calls |
| Encoding | Sanger / Illumina 1.9 |
| Total Sequences | 15204548 |
| Sequences flagged as poor quality | 0 |
| Sequence length | 35-370 |
| %GC | 50 |

## Per base sequence quality

## Per sequence quality scores

## Per base sequence content

## Per sequence GC content

## Per base N content

## Sequence Length Distribution

## Sequence Duplication Levels

## Overrepresented sequences

| Sequence | Count | Percentage | Possible Source |
| --- | --- | --- | --- |
| ACACTGAGGACTCTGTTCCTCCCCTTTCCGCCTAGGGGAAAGTCCCCGGA | 290426 | 1.910125838663537 | No Hit |
| GGGAATGTCTACGTGCGTATGCACGTGGCACTCTCTGCCCGAGGTCCGGG | 278999 | 1.8349706942948913 | No Hit |
| GGGCCACGAGCTGAGTGCGTCCTGTCACTCCACTCCCATGTCCCTTGGGA | 147843 | 0.9723603753298027 | No Hit |
| AAGCTCAGGGAGAGCCCTGTTAGGGCCGCCTCTGGCCCTAGTCTCAGACC | 137358 | 0.9034007456190083 | No Hit |
| GCTGCTACAGGAGAATAGCAGACAGGTATAGTTAAGAAACAACTTTATTT | 127572 | 0.8390384245556002 | No Hit |
| ACCAATGTCATAGGGTGCAATATCTACAATAGGTAGTCTCACAGCCTTGC | 120612 | 0.793262647465745 | No Hit |
| TTTACACCTGCTAGGGTGTTCAAAGGTCAGTGCTATAGAAATTCAGTATC | 101202 | 0.665603476012572 | No Hit |
| CTAAGTGCTGTGTTGTCGTTCCCCCTGCTTAAAATAAAGTTGTTTCTTAA | 96219 | 0.6328303873288439 | No Hit |
| CAATATCAAGGAAGGGCAGGCGTGATGGCTTATTTGTTTTGTATTCAATG | 65585 | 0.43135119833881286 | No Hit |
| TGGTTGCACGAAACACACTGGGGAATGGAGCAAAACAGTCTTTGAATATC | 60511 | 0.3979796045235939 | No Hit |
| AAAGCCAAGAAAACCAACGATGCCAGATACTGAATTTCTATAGCACTGAC | 56160 | 0.36936316686296755 | No Hit |
| TTCTTGTAGATGGCTGCTCTAAAAAGACAAATGAATGGGGAAAGACAATC | 53967 | 0.3549398508919831 | No Hit |
| TCTGCAATGCAATCACAATGCCCAAACTAGACCTGCCATTTCTCACACAC | 51695 | 0.33999695354311094 | No Hit |
| AGAGCATGACCGATGGATTCCAGTTCGAGTATGGCGGCCAGGGCTCCGAC | 46228 | 0.3040406067973872 | No Hit |
| TGGCTCTCCCAGAGGCAGAAGGACAATGGCTGTTTCAGGAGCTCTGGGTC | 45766 | 0.30100204228366406 | No Hit |
| TCTGTCTTCTTCAGTTTCGACTTATCGAATTTCTCGATCTCAGCCATATC | 44375 | 0.29185346384516003 | No Hit |
| TGTTGTGCTAAACGCTGGGAGGGTACAAGTTGGTCATTCCTAAATCTGTG | 39387 | 0.2590474902640973 | No Hit |
| ATTCTTCCTACCCATGAGCATGGAATGTTCTTCCATTTGTTTGTATCCTC | 37235 | 0.2448938304512571 | No Hit |
| TTTTTGTTGCCTGTAAAGTGCTCTGGCATTGCCTGAGGATAGATGAGAAA | 36728 | 0.24155930186152197 | No Hit |
| GAGAGGGTCACTTCATCTTCTACTCCTCCCTTTATGGCATTGTTGAGCAG | 35570 | 0.23394315963881332 | No Hit |
| TGGTTCCTGGCAAGCCCATGTGTGTTGAGAGCTTCTCAGACTATCCACCT | 32224 | 0.2119365863424549 | No Hit |
| ATGTTCTGGGAGGCCTCGGTGGACATCAGGCGCAGGAAGGTCAGCTGGAT | 32064 | 0.2108842696277456 | No Hit |
| ACACCCACCGCAACTGTCTGTCTCATATCACGAACAGCAAAGCGACCCAA | 30094 | 0.19792762007788722 | No Hit |
| GGTTATAATCATTGGCAATGTTGTAATCAACAACCCATGTTATTTCTACA | 27462 | 0.18061701012091907 | No Hit |
| TGAATCTTGAACTGAGTTCCACTTGTAAACTTCTTGTTTCTTGTGGTTCC | 26324 | 0.17313240748754913 | No Hit |
| AGAGGCCACAGTAAGGCCCATCCGAGGTCCTGAGGAGATCTTTTCTCTCT | 25842 | 0.1699623033844873 | No Hit |
| AGCGGTTCCGCTGCCCTGAGGCACTCTTCCAGCCTTCCTTCCTGGGCATG | 25588 | 0.1682917505998863 | No Hit |
| CACTTGGCTTCACCCGTGTGACTTTCGTAACGGGGAGAGAGAGAAAAGAT | 24961 | 0.1641679844741192 | No Hit |
| TCACACTTCATGATGGAGTTGAAGGTAGTTTCGTGGATGCCACAGGACTC | 24720 | 0.1625829324225883 | No Hit |
| TACTCGTGCGCCTCGCTTTGCTTTTCCTCCGCAACCATGTCTGACAAACC | 24614 | 0.1618857725990934 | No Hit |
| ACACTGAGGACTCTGTTCCTCCCCTTTCCGCCTAGGGAAAGTCCCCGGAC | 24249 | 0.15948517509366275 | No Hit |
| AAAACAGCGTCTTACTGGGGAGGGATATGTGCTTTCTCATCTATCCTCAG | 23981 | 0.15772254459652468 | No Hit |
| TGTTCCCTCTCCTCATGAGATTGGTGAAGAAAGTATTTGGCAAAGTTCTT | 23663 | 0.1556310651260399 | No Hit |
| GAAGACGTACCTTTGTTGGAAGACATACCTTTGTTGGAAGACGTACCTTT | 23577 | 0.15506544489188367 | No Hit |
| TCTTTCTGGCCTGGAGGCTATCCAGCGTACTCCAAAGATTCAGGTTTACT | 22229 | 0.14619967657045774 | No Hit |
| TGACTGGTGATGCTGATATTGGAGTGCATCTGAGGCTGTTGTAGAAATAA | 20954 | 0.13781402775011792 | No Hit |
| TCTTCCAGCCTACTTGTGTCTTCCAACAAAGGTACGTCTTCCAACAAAGG | 20913 | 0.13754437159197366 | No Hit |
| TCTTCAAGGAGAACTACAAACCACTGCTCAAGGAAATAAAAGAGGATACA | 20613 | 0.1355712777518937 | No Hit |
| AGTGTGGCGATTCCTCAGGGATCTAGAACTAGAAATACCATTGACCCAGC | 20284 | 0.13340745150727268 | No Hit |
| CAGCATGATTTATAGTCCTTTGGGTATATACCCAGTAATGGGATGGCTGG | 19906 | 0.13092135326877194 | No Hit |
| TGTCCATGTCTTACTACTTTGACCGCGATGATGTGGCTTTGAAGAACTTT | 19096 | 0.12559399990055609 | No Hit |
| TGCGAAAAGTTACTTTCAATGGACTGAACCAGATGATTGTCATAGAACTG | 18817 | 0.12375902262928172 | No Hit |
| CAGAAGCTCTTTATGTCAAACTTCTCAAGTTGATTGACCTCCAGGTAATA | 17898 | 0.11771477849917011 | No Hit |
| AACCCGCGTGCAACCTGTCCCGACTCTAGCCGCCTCTTCAGCTCGCCATG | 17091 | 0.11240715606935503 | No Hit |
| TGCATATACTGGTTAGTGAGGTGAGCCTGGCGCTCTTCTTTGCGCTGAGC | 16657 | 0.10955274698070605 | No Hit |
| CAGGAAAGGCTTAAGAGAGATATCTCCGTCACAAGCCTTTGCCATCTCCG | 16181 | 0.10642210475444583 | No Hit |
| CCCTGTTCCTACTCTCTCACTCCAGTTACCACAATTTCTCTCCAGCAACT | 15224 | 0.10012793540459079 | No Hit |

## Adapter Content

Produced by FastQC (version 0.11.7)
